# C reactive protein elevation among children or among mothers’ of children with autism during pregnancy, a review and meta-analysis

**DOI:** 10.1186/s12888-020-02619-8

**Published:** 2020-05-24

**Authors:** Rashid Nadeem, Tamseela Hussain, Hassan Sajid

**Affiliations:** 1 Dubai Hospital, Dubai, UAE; 2 Ronin Institute, 127 Haddon Pl, Montclair, NJ- 07043 USA; 3 McMaster University, Hamilton, Ontario Canada

**Keywords:** ASD, Autism, Autism spectrum disorder, CRP, Maternal CRP

## Abstract

**Objective:**

To evaluate if children with ASD, or mothers of ASD children have elevated CRP during pregnancy.

**Background:**

Autism spectrum disorder (ASD) is a neuro developmental disorder with incidence of 1 in 68 children occur in all racial, ethnic, and socioeconomic groups. Economic burden between $11.5 billion - $60.9 billion and family average medical expenditures of $4110–$6200 per year. Conflicting evidence exist about role of maternal CRP during pregnancy with ASD child.

**Methods:**

Searches on database; Pubmed, Medline, Embase and google scholar using key words; C reactive protein (CRP), Maternal CRP, ASD, autism, autistic disorder, Inflammation. All English-language studies published between 1960 and 2019 pertaining to CRP and ASD. All Studies which provided data on CRP levels during pregnancy (mCRP) of Mothers of offsprings with ASD and (mCRP) of mothers of normal subjects were selected. Data were extracted in the form of odd ratios of having high mCRP in mothers of children with ASD versus mCRP of mothers of normal controls. Since these odd ratios were adjusted, therefore no Meta regression were attempted. Significant heterogeneity was found; therefore, random effect model was employed.

**Results:**

Review of CRP levels in children with ASD showed higher level in children with ASD than control, although different methodology and absence of numerical data did not allow metanalysis. Regarding mCRP and ASD, three studies were identified that provide data on mCRP and ASD. Four datasets were created from these 3 studies as the study by Zerbo et al. provided data in 2 subsets. Total number of subjects were 5258 (Brown, *N* = 677, Zerbo = 416, Koks = 4165) extracted data from these studies was pooled for analysis. Random effect model was employed and substantial heterogeneity among the studies was observed 11. Mothers of children with ASD have adjusted Odd ratio of 1.02 (0.948 to 1.103, I2 = 75, *P* = 0.558) to have high mCRP comparing mothers of control.

**Conclusion:**

Mothers of children with ASD appear not to have elevated CRP during pregnancy. Children with ASD appear to have higher levels of CRP levels.

## Background

Autism spectrum disorder (ASD) is a Neurodevelopmental disorder that includes a wide range, “a spectrum,” of symptoms and levels of disability including social, communication and interaction challenges, and repetitive behaviors. Some subjects mildly impaired by their symptoms, lead a normal life with less dependence, while others are severely disabled and are completely dependent on their caretakers [1].

About 1 in 68 children suffer from ASD. ASD is reported to occur in all racial, ethnic, and socioeconomic groups. ASD is 5 times more common among boys (1 in 42) than among girls (1 in 189) [1]. The total costs per year for children with ASD in the United States were estimated to be between $11.5 billion - $60.9 billion (2011 US dollars) and family average medical expenditures of $4110–$6200 per year. This socioeconomic burden represents a variety of direct and in-direct costs, from medical care to special education to lost parental productivity [1].

The rising prevalence, coupled with the emotional and financial impact on the affected families, underscores the need for large, prospective, population-based studies with the goals of finding the cause, which can lead to impactful interventions and outcomes [2, 3]. Perhaps, the increase in the number of autism diagnoses that we have seen over the last decade can be explained through societal changes rather than an increase in underlying biological/environmental factors [4].

Researchers have studied various pathologic processes and factors that may be associated with ASD, with the hope to identify targets to detect patients at risk or prevent the disease by modifying risk factors for the ASD. Such studies were focused on genome in the past [5], more recently specific single-nucleotide polymorphisms (SNPs) studies are also being performed. A recent study found oxytocin receptor gene (OXTR) to be associated with ASD [6]. Nutritional deficiency as a cause or risk for ASD has been studied. Chen et al. found that mothers of ASD children had lower maternal serum levels of vitamin D and with increasing levels of vitamin D during pregnancy, there is decreasing severity of ASD. Moreover, mothers with lower D have increased odd of ASD diagnosis in offsprings [7].

Other studies have found association between environmental factors, activated immune responses in mother [8], congenital infections [9] maternal infections [10] and immune activation suggested by cytokine levels [11]. No specific pattern has been identified, although, proinflammatory and anti-inflammatory cytokines balance particularly at maternal and fetus interface has been studied [12]. Additionally, maternal antibodies and autoantibodies has also been linked to ASD [13]. Most of these antibodies are possibly directed at neuronal tissue [14]. Antibodies reactive to specific proteins were also found to be present in mothers of ASD children [15]; authors suggests them as anti-fetal brain antibodies. These antibodies are non-specific, and are also found in other disorders like multiple sclerosis [16] or schizophrenia [17] . Nonspecific symptomatology also exists across different disorders for similar behavior profile. A meta-analysis of 24 studies with ASD and 22 studies of anorexia nervosa found that both groups exhibit difficulties in set-shifting measured by the Wisconsin Card Sorting Test in the same way, which suggest complexity of the pathogenic mechanisms [18].

Multiple studies have investigated the association between C reactive protein (CRP) and ASD. The CRP is a stable protein indicating inflammation in many systemic disorders. Its concentration directly relates to the level of active inflammation in many disorders [19].

Studies on CRP have provided conflicting evidence. Some studies found that CRP is associated with pathology, particularly psychopathology, while other studies suggested otherwise. For example, CRP has been found to be associated with cognitive impairments in adults with psychiatric disorders. Cullen et al. investigated the association of salivary CRP and cognitive function in children aged 11–14 years and found that elevated salivary CRP is associated with poorer cognitive function in early life. But this association is not moderated by concurrent psychopathology [20]. Conversely, Sorokin et al. investigated umbilical cord serum concentration of CRP, and found elevated levels associated with chronic lung disease and preterm birth but no association with poor neurodevelopmental outcomes [21].

A systemic review and Metaanalysis was approached on the possible occurrence of raised CRP in subjects with ASD or in their mothers; maternal CRP (mCRP).

### Review of studies with CRP in subjects with a diagnosis of ASD

Proteomics is the large-scale study of proteomes. A proteome is a set of proteins produced in an organism, system, or biological context. Such studies have found higher levels of CRP in children with ASD and other neuropsychiatric disorders, in parallel to immunoglobulins irregularities [22–24]. Chaudhary et al. found significantly elevated serum CRP levels in subjects with ASD compared to control [22]. While Ramsey et al. reported the complex interaction of CRP and age as CRP levels were only 50% of controls in subjects younger than 9 years of age, while CRP levels in subjects with ASD were increased to more than 200% of controls in children above 13 years of age [23].

Khakzad et al.^25^ reported a higher mean concentration of CRP in ASD versus the controls. The mean concentration of high sensitivity (hs)-CRP in children with ASD (540.1 ± 1125.5 ng/ml) was significantly (*P* < 0.0001) higher than control group (1.3 ± 1.0 ng/ml). In severe ASD, the mean level of hs-CRP (985.1 ± 1432.1 ng/ml) was significantly (*P* = 0.008) higher than the mean level of hs-CRP in patients with mild-to-moderate ASD (147.1 ± 60.4 ng/ml). There was a positive correlation between hs-CRP concentration and ASD severity (r = 0.34; *P* = 0.039). These findings suggest role of inflammation in ASD [25].

Metaanalysis of these studies was not possible as the required data were not provided by authors owing to the different design and methods of studies. Conversely, studies on mCRP have provided data that can be analyzed by metaanalysis.

## Metaanalysis of maternal CRP during pregnancy (mCRP) and ASD

### Methods

We followed PRISMA guidelines [26, 27] to perform data review and metaanalysis.

### Rationale

Multiple studies available aiming to address the association between mCRP and ASD reported conflicting evidences.

### Objective

To detect association between mCRP and child’s diagnosis of ASD by collecting and analyzing all available observational or cross sectional non interventional studies where levels of mCRP were compared between mothers of children without ASD versus mCRP of mothers of children with ASD.

**Fig. 1:**
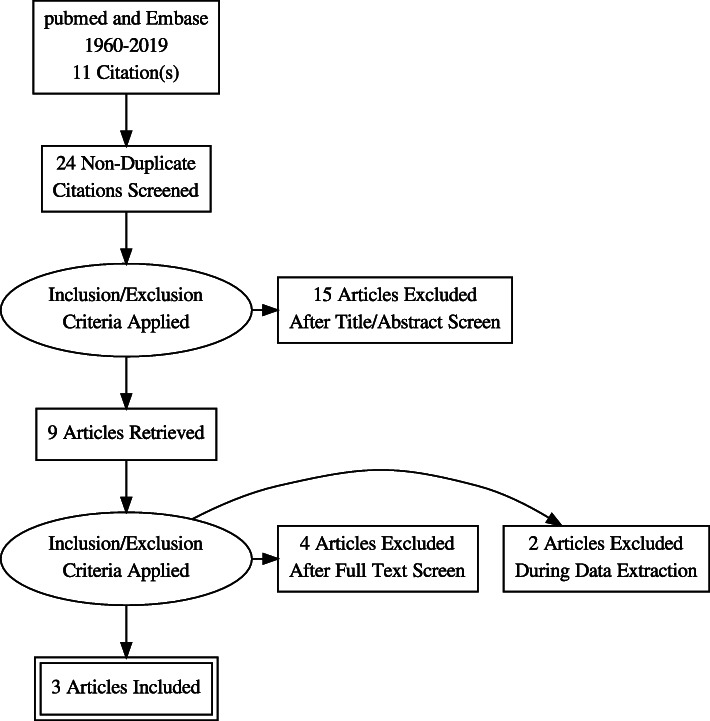
Prisma flow diagram for searching articles

Data searches were performed on Pubmed, Embase and Google scholar using key words; C reactive protein (CRP), Maternal CRP, ASD, autistic disorder, Inflammation and combination of these. Initial screening reveal (Autism Spectrum Disorder = 27,011, CRP = 74,382, Maternal C reactive protein = 42, but focused searches narrowed down to relevant articles for example (“Autism Spectrum Disorder”[Mesh]) AND “C-Reactive Protein”[Mesh] =11. PRISMA flow diagram (Fig. 1).

### Inclusion criteria

All English-language studies published between 1960 and 2019 pertaining to CRP and ASD. Studies which provided data on CRP levels during pregnancy (mCRP) of Mothers of offspring with ASD and (mCRP) of mothers of children without ASD were selected. Data was extracted in the form of odd ratios of having high mCRP in mothers of children with ASD versus mCRP of mothers of children without ASD. Since these odd ratios were already adjusted, therefore no Meta regression analysis were attempted. Extracted data was analyzed by Comprehensive meta-analysis software (Biostat, Englewood, NJ, USA). Significant heterogeneity was found; therefore, random effect model was employed.

## Results

**Fig. 2:**
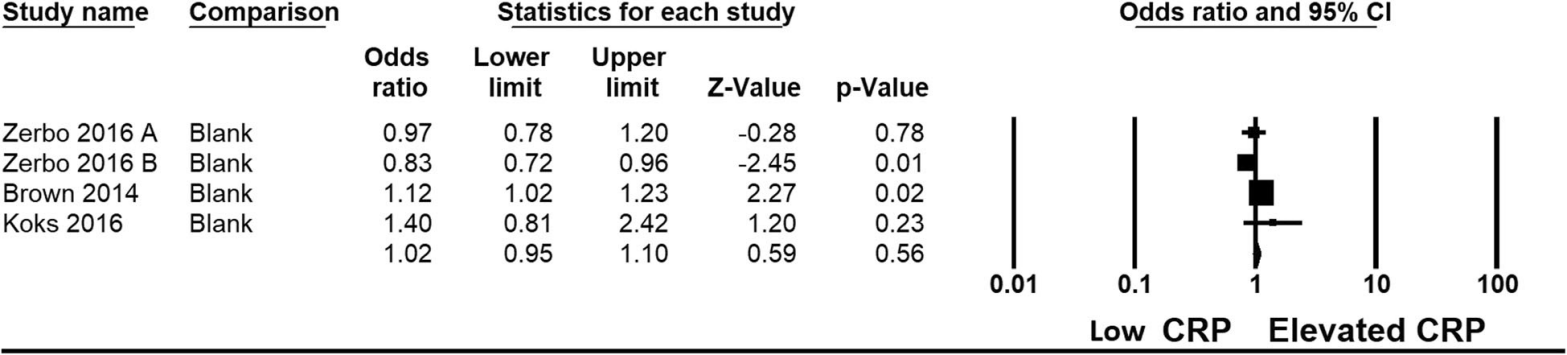
Forest plot of studies mCRP and ASD

Studies screening and selection strategy is outlined in Fig. 1. Three studies [10, 28, 29] (Table 1) were identified, meeting the inclusion criteria. Four datasets were created from these 3 studies as the study by Zerbo et al. provided data in 2 subsets [10]. Total number of subjects were 5258 (Brown, *N* = 677, Zerbo = 416, Koks = 4165) extracted data from these studies were pooled for analysis. Subjects composition for each study as follows; for Brown et al. CRP modeled as a continuous variable in relation to risk of childhood ASD in offspring. The analysis revealed a significant association between increasing maternal CRP and risk of ASD (OR = 1.12, 95% CI = 1.02–1.24, *p* = 0.02 while their quintile analysis compares fifth quintile CRP value (N 164 mothers) to first quintile CRP Value (N 119 mothers) for Odds of ASD 1.43 95% CI =1.02–2.01, *p* = 0.03. Zerbo et al. compared 500 mothers of children with ASD, 235 mothers of children with developmental delay and 580 mothers of children from general population. Koks et al. had total sample of 4165 mothers, group with highest per quartiles were compared with group with lowest quartile value of CRP (N for each group not provided). For meta-analysis, Random effect model was used as substantial heterogeneity among the studies was observed. Mothers of children with ASD had Odd ratio of 1.02 (0.948 to 1.103, I^2^ = 75, *P* = 0.558) to have high mCRP, comparing mothers of control (Fig. 2). We acknowledge that number of studies are small and metaanalysis is apparently dominated by one large study.

**Table 1:** Characterstics of included studies with outcomes

Study Authors, year	Study Sample characteristics;	findings	outcomes	Critical review
Brown et al. 2014 (Finland)	−677 mothers of ASD were matched with 677 control. -Covariates included maternal age, paternal age, number of previous births, maternal socioeconomic status, pre-term birth, low birthweight, maternal/ parental history of psychiatric disorders, and gestational week of the blood draw	80% increase in risk of ASD with elevated mCRP, in the highest decile (> 9.55 mg/dl), compared to the lowest decile (0.10–0.57 mg/dl) (OR = 1.80, 95% CI = 1.09–2.97, *p* = .02) greater association for females, but no interaction between mCRP and gender or mental retardation.	**significant association** between increasing maternal CRP (as continuous variable) and risk of ASD (OR = 1.12, 95% CI = 1.02–1.24, *p* = 0.02	-Retrospective Case control study provides only weak evidence for association. − 455 cases were excluded from sample of 1132). -Many important confounding factors were not recorded i.e. history of infection, or inflammatory disorder, genetic analysis, immunoglobulin levels, psychopathology in pregnancy, or ethnicity.
Koks et al. 2016 (Netherlands)	-mCRP levels in early pregnancy and Social Responsiveness Scale (SRS) scores in children at the age of 6 years were analyzed for 4165 mother-child pairs.	mCRP levels and SRS scores were found to be associated but sequential modelling with confounding factors made the association disappear.	**no association** between elevated levels of mCRP during early pregnancy and parent-reported autistic traits in children at the age of 6 years.	-Retrospective Case and control study provide Weak evidence. − 1042 mother-child pairs data excluded have younger mothers with different lifestyle i.e. more smoking.
Zerbo 2016 (USA)	-population-based nested case–control study with 500 children with ASD, 235 with developmental delay (DD) and 580 general population (GP) − 2 phase study.	-First sample set. Median mCRP levels were lower in the mothers of children with ASD (1.28 mg/dl, interquartile range = 0.54–3.06) compared with the mothers of GP controls (2.43 mg/dl, interquartile range = 0.61–3.82 -Median CRP levels were significantly lower in the mothers of children with ASD (1.94 mg/dl, interquartile range = 1.04–3.90) compared with the mothers of GP controls (2.40 mg/dl, interquartile range = 1.32–4.48	mCRP levels in mid-pregnancy were lower in mothers of ASD compared with controls. The maternal CRP levels in the upper third and fourth quartiles were associated with a 45 and 44% decreased risk of ASD, respectively (**negative association)**	-Retrospective case control study. -Study has 2 phases with different sample size and different technique for mCRP measurement.

## Discussion

Our results indicate that there is no association between the levels of maternal CRP and ASD. More likely reason for this result is small number of studies with variable methodology not enough to exclude this possibility or estimate the association of CRP in ASD.

Maternal CRP association with ASD is confounded by multiple factors i.e. age, ethnicity, smoking habits of mothers, socioeconomic status, and educational status. CRP is nonspecific, and can be elevated in many disorders from acute cardiovascular disease [30] to chronic obstructive sleep apnea [31]. Moreover, its level also changes with time and severity of ongoing disease process, therefore one-point testing of mCRP level or ASD subjects is not enough, which is the case in most studies.

Prospective studies with long term follow up with better methodology, are required to assess if CRP is associated with ASD. Such studies are difficult as factors involved are with both subjects (mother and child) and confounding factors are in perinatal span over long period of time. Consequently, there is no convincing evidence available so far for any specific pattern or elevated level of CRP in ASD children or their mothers. Role of CRP or inflammation cannot be excluded as well, as studies suggesting role of inflammation and immunoglobulins continue to be reported [32]. Another study suggests children with ASD have higher plasma levels of glutamate, which may have a contributory role in pathogenesis of ASD [33].

It appears pathogenesis of ASD is multifactorial and interaction among all contributing factors is complex. We have no convincing data thus far about position of mCRP or child CRP. A genetic predisposition or nutritional deficiency which may be compounded by an infection or inflammation during pregnancy, may modulate neurodevelopmental processes of the fetus with additional post birth factors from environment, nutrition or habits like smoking by mother may dysregulate immune system or nervous system growth and increased risk of ASD [34].

### Clinical implications and recommendations

Future research studies are needed to evaluate these risk factors by better methodology and long term follow up. These studies should be planned for at risk population prenatally, and should be performed with multidisciplinary approach where obstetrician, gynecologist, pediatrician, psychologist, psychiatrist, nutritionist and trialist are team members, and multiple measurements for these risk factors are recorded and subjects are followed up for longer duration. If such a correlation is established for mCRP with ASD it may help identify women at risk for giving birth to children with ASD. Subsequent studies to probe reasons for this elevation can help define etiology. It is also important for future studies to involve treatment measures to improve CRP and observe if it affects occurrence of ASD or severity of symptoms of ASD.

### Study limitations

**Fig. 3:**
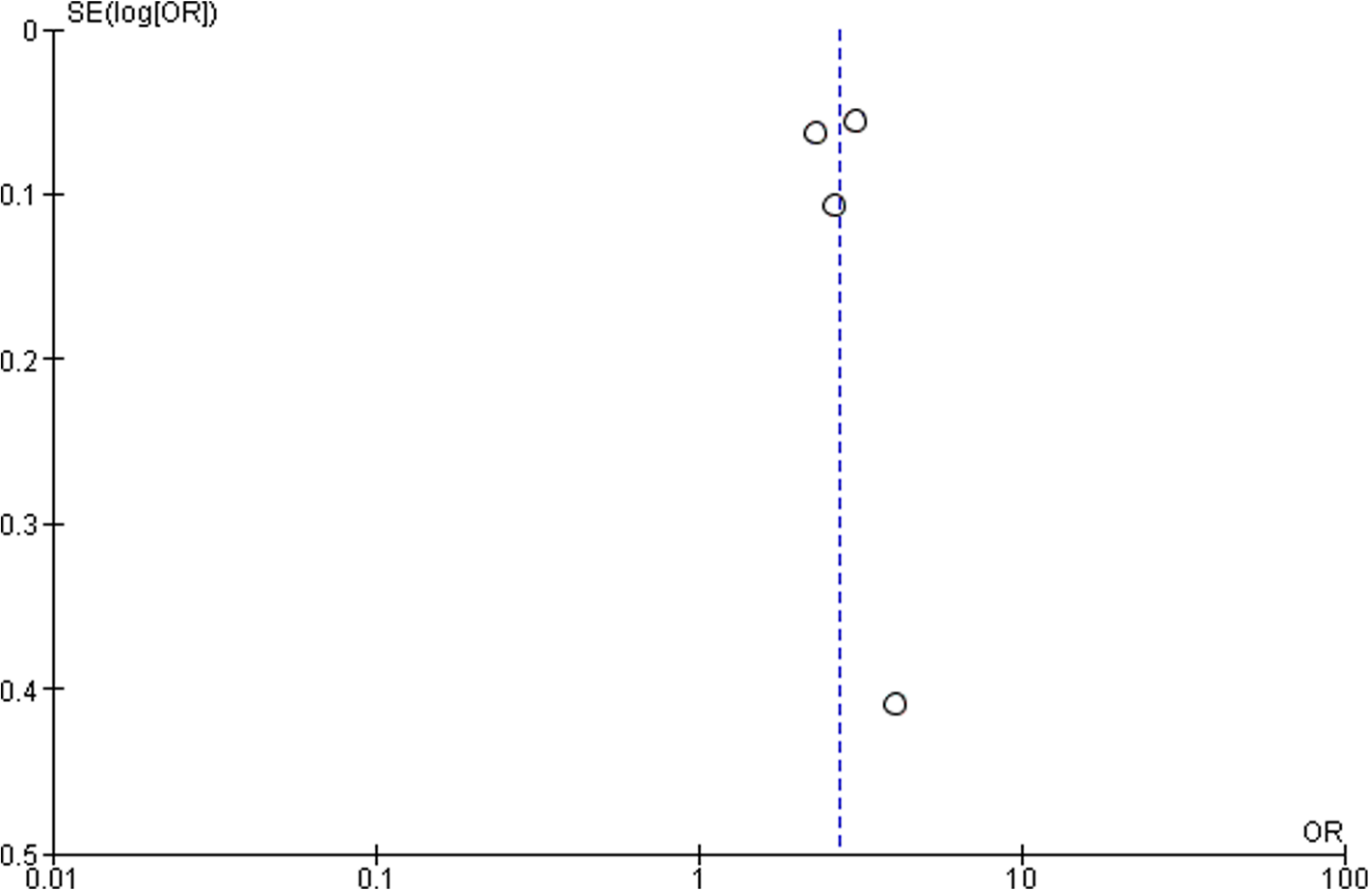
Funnel plot for publication bias

This metanalysis was only from 3 studies, which was heterogeneous. There are no more publications available with the data on this issue. All the studies are retrospective, observational and performed in different populations with different methodology which provides weak level of evidence. Data available are so sparse, that it cannot exclude the role of CRP in ASD as well. Random effect model was employed to address heterogeneity. Study of confounding factors by metaregression, a powerful mathematical technique and subgroup analysis helps in multifactorial disorders, but this small data are insufficient to perform Metaregression, to address the effect of confounding factors. Moreover, data provided in these studies were already adjusted for these variables. This study only highlights the fact that data available on this issue are only minimum and more studies are urgently needed. Funnel plot (Fig. 3) was also generated to estimate bias although small number of studies preclude reliable estimate for bias, which suggests publication bias may be present.

## Conclusion

It appears that presently available data are insufficient to identify or exclude CRP role in ASD. Studies are urgently needed on this very important clinical question which may help us identify a mechanism and population at risk, which could guide us to the etiology of ASD and help us provide better management or prevention of ASD.

## Data Availability

not applicable.

## Abbreviation

ASD: Autistic spectrum disorder
CRP: C Reactive protein
mCRP: Maternal C reactive protein
SNP: Specific single-nucleotide polymorphisms
OXTR: Oxytocin receptor gene

## References

[1] Baio J, Wiggins L, Christensen DL (2018). Prevalence of autism spectrum disorder among children aged 8 years — autism and developmental disabilities monitoring network, 11 sites, united states. MMWR.

[2] Zuckerman KE, Lindly OJ, Bethell CD, Kuhlthau K (2014). Family impacts among children with autism spectrum disorder: the role of health care quality. Acad Pediatr.

[3] Gardener H, Spiegelman D, Buka SL (2009). Prenatal risk factors for autism: comprehensive meta-analysis. Br J Psychiatry.

[4] Fombonne Eric (2003). The Prevalence of Autism. JAMA.

[5] Abrahams BS, Geschwind DH (2008). Advances in autism genetics: on the threshold of a new neurobiology. Nat Rev Genet.

[6] LoParo D, Waldman ID (2015). The oxytocin receptor gene (OXTR) is associated with autism spectrum disorder: a meta-analysis. Mol Psychiatry.

[7] Chen J, Xin K, Wei J, Zhang K, Xiao H (2016). Lower maternal serum 25 (OH) D in first trimester associated with higher autism risk in chinese offspring. J Psychosom Res.

[8] Shi L, Fatemi SH, Sidwell RW, Patterson PH (2003). Maternal influenza infection causes marked behavioral and pharmacological changes in the offspring. J Neurosci.

[9] Chess S (1971). Autism in children with congenital rubella. J Autism Child Schizophr.

[10] Zerbo O, Traglia M, Yoshida C (2016). Maternal mid-pregnancy C-reactive protein and risk of autism spectrum disorders: the early markers for autism study. Transl Psychiatry.

[11] Boulanger LM, Shatz CJ (2004). Immune signalling in neural development, synaptic plasticity and disease. Nat Rev Neurosci.

[12] Meyer U, Murray PJ, Urwyler A, Yee BK, Schedlowski M, Feldon J (2008). Adult behavioral and pharmacological dysfunctions following disruption of the fetal brain balance between pro-inflammatory and IL-10-mediated anti-inflammatory signaling. Mol Psychiatry.

[13] Braunschweig D, Ashwood P, Krakowiak P (2008). Autism: maternally derived antibodies specific for fetal brain proteins. Neurotoxicology..

[14] Dalton P, Deacon R, Blamire A (2003). Maternal neuronal antibodies associated with autism and a language disorder. Ann Neurology.

[15] Croen LA, Braunschweig D, Haapanen L (2008). Maternal mid-pregnancy autoantibodies to fetal brain protein: the early markers for autism study. Biol Psychiatry.

[16] Ryberg B (1982). Antibrain antibodies in multiple sclerosis: relation to clinical variables. J Neurol Sci.

[17] Libbey JE, Fujinami RS (2010). Role for antibodies in altering behavior and movement. Autism Res.

[18] Westwood H, Stahl D, Mandy W, Tchanturia K (2016). The set-shifting profiles of anorexia nervosa and autism spectrum disorder using the Wisconsin card sorting test: a systematic review and meta-analysis. Psychol Med.

[19] Castro C, Gourley M (2010). Diagnostic testing and interpretation of tests for autoimmunity. J Allergy Clin Immunol.

[20] Cullen AE, Tappin BM, Zunszain PA (2017). The relationship between salivary C-reactive protein and cognitive function in children aged 11–14 years: does psychopathology have a moderating effect?. Brain Behav Immun.

[21] Sorokin Y, Romero R, Mele L (2014). Umbilical cord serum interleukin-6, C-reactive protein, and myeloperoxidase concentrations at birth and association with neonatal morbidities and long-term neurodevelopmental outcomes. Am J Perinatol.

[22] Chaudhry M, Shahzad F, Aziz S (2015). Serum immunoglobulins and CRP levels in autistic children. Biomedica..

[23] Ramsey JM, Guest PC, Broek JA (2013). Identification of an age-dependent biomarker signature in children and adolescents with autism spectrum disorders. Molecular autism.

[24] Mitchell RH, Goldstein BI (2014). Inflammation in children and adolescents with neuropsychiatric disorders: a systematic review. J Am Acad Child Adolesc Psychiatry.

[25] Khakzad MR, Javanbakht M, Shayegan MR (2012). The complementary role of high sensitivity C-reactive protein in the diagnosis and severity assessment of autism. Res Autism Spectr Disord.

[26] Liberati A, Altman DG, Tetzlaff J (2009). The PRISMA statement for reporting systematic reviews and meta-analyses of studies that evaluate health care interventions: explanation and elaboration. PLoS Med.

[27] Liberati A, Altman D, Tetzlaff J, et al. The PRISMA statement for reporting systematic reviews and meta-analyses of studies that evaluate healthcare interventions. BMJ. 2009;339. 10.1136/bmj.b2700 PMC2714672 19622552

[28] Brown AS, Sourander A, Hinkka-Yli-Salomäki S, McKeague IW, Sundvall J, Surcel H (2014). Elevated maternal C-reactive protein and autism in a national birth cohort. Mol Psychiatry.

[29] Koks N, Ghassabian A, Greaves-Lord K (2016). Maternal C-reactive protein concentration in early pregnancy and child autistic traits in the general population. Paediatr Perinat Epidemiol.

[30] Stumpf C, Sheriff A, Zimmermann S (2017). C-reactive protein levels predict systolic heart failure and outcome in patients with first ST-elevation myocardial infarction treated with coronary angioplasty. Arch Med Scie.

[31] Nadeem R, Molnar J, Madbouly EM (2013). Serum inflammatory markers in obstructive sleep apnea: A meta-analysis. J Clin Sleep Med.

[32] Grether JK, Croen LA, Anderson MC, Nelson KB, Yolken RH (2010). Neonatally measured immunoglobulins and risk of autism. Autism Res.

[33] Cai J, Ding L, Zhang J, Xue J, Wang L (2016). Elevated plasma levels of glutamate in children with autism spectrum disorders. Neuroreport..

[34] Marques AH, O'Connor TG, Roth C, Susser E, Bjørke-Monsen A (2013). The influence of maternal prenatal and early childhood nutrition and maternal prenatal stress on offspring immune system development and neurodevelopmental disorders. Front Neurosci.

